# Commentary: Self-Oriented Empathy and Compassion Fatigue: The Serial Mediation of Dispositional Mindfulness and Counselor's Self-Efficacy

**DOI:** 10.3389/fpsyg.2021.665632

**Published:** 2021-05-17

**Authors:** Helen R. Chapman

**Affiliations:** School of Psychology, University of Lincoln, Lincoln, United Kingdom

**Keywords:** compassion fatigue, Interpersonal Reactivity Index, self-oriented empathy, personal distress, empathy

Zhang et al. (2021), in their recent article exploring “the association between self-oriented empathy and compassion fatigue” (p. 1) stated that

“Compassion fatigue (CF) is an empathetic reaction resulting from frequently witnessing the emotional or physical suffering of others or repeatedly listening to a person suffering from mental or physical dysfunction” (p. 1).

The authors measured “self-oriented empathy” using Davis' original Interpersonal Reactivity Index (IRI) scale (Davis, 1983) and stated,

“The personal distress subscale measures self-oriented empathy, that is, distress and discomfort elicited by witnessing another person's suffering. High scores of the personal distress subscale indicate a high tendency to experience self-oriented empathy when observing the suffering of others” (p. 4).

If one returns to Davis' original Interpersonal Reactivity Index (IRI) scale development (Davis, 1983), the following is stated about the subscales under consideration,

“The Empathic Concern (EC) scale assesses “other-oriented” feelings of sympathy and concern for unfortunate others, and the Personal Distress (PD) scale measures “self-oriented” feelings of *personal anxiety and unease* in tense interpersonal settings” (p. 114) (emphasis added).

The Personal Distress subscale (Davis, 1980) contains items such as

Being in a tense emotional situation scares me.

I am usually pretty effective in dealing with emergencies (-).

I tend to lose control during emergencies.

When I see someone who badly needs help in an emergency, I go to pieces (p. 96).

These items do not reflect “empathy” or “self-oriented empathy.” Empathy is defined in many ways throughout the literature, but is generally considered to have two components; cognitive empathy (intellectually understanding another person's emotions and perspective) and affective or emotional empathy (being affected by and sharing another's emotions, both positive and negative). In other words, empathy relates to feeling with another. Empathy is necessary for empathic concern and compassion (wishing to or acting to alleviate suffering) which are other-oriented; feeling for another (Klimecki and Singer, 2011; Strauss et al., 2016). Decety (2020), reviewing the use of the term “empathy” in medicine states,

“Empathy is a broad construct that refers to the ability to sense other people's emotions, coupled with the ability to imagine what someone else might be thinking or feeling” (p. 563).

The author is unaware of any published definition of the concept of “self-oriented empathy” which appears to be a new term coined by Zhang et al. It should not be confused with “self-compassion” (Neff, 2003) which is associated with lower levels of both burnout (Gerber and Anaki, 2020; Hashem and Zeinoun, 2020) and secondary traumatic stress (Neff et al., 2020).

Compassion fatigue is defined by Stamm (2010) as having 2 components.

“The first part concerns things such like exhaustion, frustration, anger and depression typical of burnout. Secondary Traumatic Stress is a negative feeling driven by fear and work-related trauma. Some trauma at work can be direct (primary) trauma. In other cases, work-related trauma be a combination of both primary and secondary trauma” (p. 8).

Decety (2020) summarizes this as,

“Compassion fatigue is the physical and mental exhaustion and emotional withdrawal experienced by individuals who care for sick or traumatized people over an extended period of time” (p. 563).

In a very recent publication (Eng et al., 2021) which validated a new measure, The Compassion Fatigue Inventory, three factors were found to contribute to compassion fatigue:

1. Reduced Compassion—My will to help has declined; I feel irritated when patients complain
2. Social Life—I have started to withdraw from social interactions; I have noticed that my patience in personal relationships has dwindled
3. Workplace—I feel that my workplace provides care that is in accordance with my values (rev) (p. 13)

It did not include any items appearing to measure what one could consider to be self-oriented empathy or compassion. The authors concluded that,

“Even though Compassion fatigue had a high correlation with both burnout and STS [Secondary Traumatic Stress], the results suggest a narrower conceptualization of compassion fatigue” (p. 1).

Therefore, it can be seen that personal distress is just that, personal distress, and not self- oriented empathy. In other words, it reflects a tendency to be emotionally overwhelmed because of poor emotional regulation of negative affective empathy (Hofmeyer et al., 2019), inadequate self-other differentiation (Klimecki and Singer, 2011), or the nature of top-down control not/used such as cognitive appraisal of the situation (Lamm et al., 2007a,b). This leads to the argument that compassion fatigue should be called “Empathic distress fatigue” (Klimecki and Singer, 2011; Hofmeyer et al., 2019) where high levels of inadequately modulated empathy for distress, or a lack of ability to respond prosocially with compassion (Duarte and Pinto-Gouveia, 2017), result in personal distress and *self-oriented attempts to reduce one's own suffering*. This is apparently different to being overwhelmed to the point of coping badly as implied by the nature of the IRI items.

Zhang et al. (2021) also found that, “Self-oriented empathy was positively associated with compassion fatigue” (p. 1). This makes absolute sense; *personal distress*, as measured by the IRI, not self-oriented empathy, is associated with compassion fatigue.

In summary, the use of the term “self-oriented empathy” in this paper (Zhang et al., 2021) is confusing and the paper would be enhanced if it were re-written to reflect the fact that it is personal distress that is associated with compassion fatigue.

## Author Contributions

The author confirms being the sole contributor of this work and has approved it for publication.

## Conflict of Interest

The author declares that the research was conducted in the absence of any commercial or financial relationships that could be construed as a potential conflict of interest.
